# Topographic Reconfiguration of Local and Shared Information in Anesthetic-Induced Unconsciousness

**DOI:** 10.3390/e20070518

**Published:** 2018-07-10

**Authors:** Heonsoo Lee, Zirui Huang, Xiaolin Liu, UnCheol Lee, Anthony G. Hudetz

**Affiliations:** 1 Department of Anesthesiology, Center for Consciousness Science, University of Michigan Medical School, Ann Arbor, MI 48109, USA; 2 Department of Radiology, Medical College of Wisconsin, Milwaukee, WI 53226, USA

**Keywords:** permutation entropy, functional magnetic resonance image, mutual information, functional connectivity, anesthesia, consciousness

## Abstract

Theoretical consideration predicts that the alteration of local and shared information in the brain is a key element in the mechanism of anesthetic-induced unconsciousness. Ordinal pattern analysis, such as permutation entropy (PE) and symbolic mutual information (SMI), have been successful in quantifying local and shared information in neurophysiological data; however, they have been rarely applied to altered states of consciousness, especially to data obtained with functional magnetic resonance imaging (fMRI). PE and SMI analysis, together with the superb spatial resolution of fMRI recording, enables us to explore the local information of specific brain areas, the shared information between the areas, and the relationship between the two. Given the spatially divergent action of anesthetics on regional brain activity, we hypothesized that anesthesia would differentially influence entropy (PE) and shared information (SMI) across various brain areas, which may represent fundamental, mechanistic indicators of loss of consciousness. FMRI data were collected from 15 healthy participants during four states: wakefulness (W), light (conscious) sedation (L), deep (unconscious) sedation (D), and recovery (R). Sedation was produced by the common, clinically used anesthetic, propofol. Firstly, we found that that global PE decreased from W to D, and increased from D to R. The PE was differentially affected across the brain areas; specifically, the PE in the subcortical network was reduced more than in the cortical networks. Secondly, SMI was also differentially affected in different areas, as revealed by the reconfiguration of its spatial pattern (topographic structure). The topographic structures of SMI in the conscious states W, L, and R were distinctively different from that of the unconscious state D. Thirdly, PE and SMI were positively correlated in W, L, and R, whereas this correlation was disrupted in D. And lastly, PE changes occurred preferentially in highly connected hub regions. These findings advance our understanding of brain dynamics and information exchange, emphasizing the importance of topographic structure and the relationship of local and shared information in anesthetic-induced unconsciousness.

## 1. Introduction

The alteration of temporal dynamics in the brain is thought to be a key element in the mechanism of anesthetic-induced unconsciousness. For instance, the anesthetic, propofol, causes regular and stereotypic dynamics [1,2], along with loss of consciousness. In the past, these changes were identified by using entropy or complexity measures. Among others, permutation entropy (PE) has been successfully applied to quantify local information derived from electroencephalographic signals (EEG), due to its robustness to artifacts, invariance with respect to nonlinear monotonous transformations, computational efficiency, and the minimal requirement for the pre-processing of EEG signals [3,4]. The PE of the EEG captures the progressive increase of regularity in close correlation with the level of consciousness under sedation [1,5,6]. It also provides reasonable pharmacokinetic-pharmacodynamic models to predict the drug concentration and its effects [4,7].

Although PE analysis has been widely used in EEG data, its application to functional magnetic resonance (fMRI) data has been scarce [8]. Applying PE to fMRI data could be beneficial because it can be used to localize brain areas where changes in temporal dynamics or local information occur, which was difficult to do with EEG due to its poor spatial resolution. In addition, PE analysis can be easily expanded to symbolic mutual information (SMI) analysis [9]. SMI analysis provides an efficient way to quantify both linear and nonlinear coupling between brain regions, in contrast to conventional functional connectivity analysis in which only linear coupling can be detected. Thus, PE and SMI quantify two aspects of neural information processing: local and shared information, respectively.

Based on former EEG investigations, we hypothesized that the anesthetic, propofol, would suppress both local (PE) and shared information (SMI) of fMRI signals. We predicted that the suppression of PE and SMI would be differential across brain regions and connections, respectively, leading to a spatial reorganization of PE and SMI. More specifically, considering the essential role of highly connected hub regions for efficient information processing, we further hypothesized that hub regions will be preferentially affected with loss and recovery of consciousness. 

To test our hypothesis, we recruited fifteen healthy participants under four conditions using fMRI: (1) wakefulness, (2) propofol-induced light sedation and (3) deep sedation, and (4) recovery. To estimate local and shared information, PE and SMI respectively were estimated from blood oxygen level-dependent (BOLD) signals of 226 functional brain areas. We found that propofol differentially affected PE and SMI across brain regions, resulting in a topographical reorganization of PE and SMI. The relationship between PE and SMI across 226 regions were significantly altered across four conditions. Moreover, brain areas in which PE changes correlated with the state of consciousness were mostly hub regions.

## 2. Materials and Methods

### 2.1. Anesthetic Protocol

The present analysis was based on data previously published [10,11,12]. Briefly, the study included 15 healthy participants (9 males/6 females; 19–35 years). The fMRI data was collected from 15-min scans in (1) wakefulness, (2) propofol-induced light sedation (~1 μg/mL plasma concentration) and (3) deep sedation (~2 μg/mL plasma concentration), and (4) recovery. In light sedation, participants showed a lethargic response to verbal commands (OAAS score 4), and in deep sedation, participants showed no response to verbal commands (OAAS score 2-1). To evaluate the OAAS score, the anesthesiologist entered the scanner room and performed the defined assessment procedures at the bedside of the scanner before the next scan, after the propofol had reached a predicted target concentration. Propofol was administered at a target plasma concentration (light sedation: 0.98 ± 0.18 μg/mL; deep sedation: 1.88 ± 0.24 μg/mL), which was maintained at equilibrium by manually adjusting the infusion rate as predicted by the software STANPUMP (Shafer, Palo Alto, CA, USA) based on the pharmacokinetic model [13]. The anesthetic equilibrium assures a steady state for 15-minute fMRI data, minimizing the interference of external conditions to low-time resolution BOLD signals.

### 2.2. fMRI Data Acquisition and Preprocessing

Resting-state functional MRI (rs-fMRI) data were collected using a 3T Signa GE 750 scanner (GE Healthcare, Waukesha, WI, USA) using gradient-echo EPI images of the whole brain (41 slices, TR/TE = 2000/25 ms, slice thickness = 3.5 mm, in-plane resolution = 3.5 × 3.5 mm; field of view (FOV) = 224 mm, flip angle = 77°, image matrix: 64 × 64). High-resolution spoiled gradient-recalled echo (SPGR) anatomical images were acquired before the functional scans.

Analysis of Functional NeuroImages (AFNI, http://afni.nimh.nih.gov/afni) was used for imaging data preprocessing: (1) discarding the first two frames of each fMRI run; (2) physiological noise correction through removal of time-locked cardiac and respiratory artifacts using RETROICOR [14]; (3) slice timing correction; (4) rigid body correction/realignment within and across runs; (5) co-registration with high-resolution anatomical images; (6) spatial normalization into Talaraich stereotactic space; (7) resampling to 3 × 3 × 3 mm^3^ voxels; (8) regressing out linear and nonlinear drift, head motion and its temporal derivative, and mean time series from the white matter (WM) and cerebrospinal fluid (CSF), and band-pass filtered to 0.01 and 0.1 Hz [15,16]. The WM and CSF masks were eroded by one voxel to minimize partial voluming with gray matter [17]; (9) spatial smoothing with 8 mm full-width at half-maximum isotropic Gaussian kernel; (10) the time-course per voxel of each run was normalized to zero mean and unit variance (*z* value), accounting for differences in variance of non-neural origin (e.g., the distance from head coil) [18,19]. 

### 2.3. Definition of Functional Networks

We adopted a well-established network template from a previous study [20], containing 264 putative functional areas (10 mm diameter spheres, 32 voxels per sphere) across the whole brain’s gray matter. The original template consisted of 11 functional networks, plus 36 uncertain areas [20,21,22]. In this study, we excluded the cerebellum and the uncertain areas, thus including 10 functional networks (226 functional areas in total); namely, the subcortical (Sub), dorsal attention (DA), ventral attention (VA), default mode (DMN), frontoparietal task control (FPTC), cingulo-opercular task control (COTC), salience (Sal), sensory/somatomotor (SS), auditory (Audi), and visual networks (Visual). The time series of the fMRI-BOLD signal was extracted from the functional mask for each area for the following measurement.

### 2.4. Ordinal Pattern Analysis

This study applies PE and SMI, which are information-theoretic measures of temporal dynamics and time-average functional connectivity, respectively. PE was estimated from each of the 226 brain areas using symbolic transformation, with pattern length m and time lag τ [3]. The ordinal patterns are described by order relations between present and equidistant past amplitude values of BOLD time series. For N-point BOLD time series, an embedded time series is first obtained, such that,

(1) $$X_{t}=\left\{x_{t},\;x_{t+\tau },\;\ldots x_{t+\left(m-1\right)\tau }\right\},\;t=1,\;2,\;\ldots ,N-\left(m-1\right)\tau ,$$

where $x_{t}$ is amplitude at time t, m is embedding dimension (pattern length), and τ is the time lag. Rearranging $X_{t}$ in ascending order yields $\left\{x_{t+\left(i_{1}-1\right)\tau }\leq x_{t+\left(i_{2}-1\right)\tau }\leq \ldots \leq x_{t+\left(i_{m}-1\right)\tau }\right\}$, where $i_{k}$ denotes the associated index of the *k*th rank. Using the Shannon entropy formula, PE is defined as,

(2) $$\mathrm{PE}\left(X\right)=-\frac{1}{\log\left(m!\right)}\underset{j}{\sum }p\left(\hat{x}\right)\log p\left(\hat{x}\right),$$

where 0 ≤ $p\left(\hat{x}\right)$ ≤ 1 is the probability of the pattern $\hat{x}$, as estimated by counting the number of times each pattern occurs in a given sample; and the normalization by log(*m*!) reflects the maximum entropy that the system has. Thus, PE has a value between 0 and 1.

Shared information among the 226 regions was estimated using SMI. As in PE, the estimation of the probability distribution for SMI is also based on the ordinal patterns. SMI is defined as,

(3) $$\mathrm{SMI}\;\left(X,Y\right)=\underset{\hat{x},\;\hat{y}}{\sum }p\left(\hat{x},\;\hat{y}\right)\log\frac{p\left(\hat{x},\;\hat{y}\right)}{p\left(\hat{x}\right)p\left(y\right)},$$

where $p\left(\hat{x},\;\hat{y}\right)$ is the joint distribution between signal X and signal Y. The 226 by 226 SMI matrix was obtained for each subject for each state. 

Considering the low temporal resolution of fMRI data, we chose the parameter τ = 1. In addition, we chose the pattern size m = 3 to avoid a sparse distribution of histogram; for instance, a joint distribution for SMI can be sparse if $N<{\left(m!\right)}^{2}$. However, using a larger pattern length (m = 4) did not yield a qualitative difference in the results.

### 2.5. Topographic Similarity and Relationship between PE and SMI

We investigated the similarities between topographic structures from different states. Spearman correlation was calculated between a reference PE vector (vectorized SMI matrix) and a PE vector (vectorized SMI matrix) for each subject and for each state. The reference PE vector (vectorized SMI matrix) represents an average of PE vectors (vectorized SMI matrices) from 15 subjects in the W state. 

Next, the relationship between PE and SMI and its changes across the four states were investigated. To compare the 226 by 1 PE vector and the 226 by 226 SMI matrix, we converted the SMI matrix into a 226 by 1 SMI vector; that is, the SMI node degree for each of the 226 areas was defined by the summation of 225 SMI values connected to an area. Here, the SMI matrix was considered as a connectivity graph. Each of 226 areas represents a node and each SMI connection represents an edge connecting the two corresponding nodes (areas) in the graph. In this analysis, a Pearson correlation between the two was calculated for each state for each subject. 

Lastly, we related PE changes and the SMI node degree of the baseline W state. In the PE analysis, we searched the brain areas whose PE significantly decreased and increased during the loss and recovery of consciousness, respectively. Next, we determined which of those brain areas had a preferential distribution of the SMI node degree. Here we used an averaged SMI node degree across 15 subjects in the baseline W state. We divided the 226 brain areas into three groups; high degree nodes (top 5% of nodes ranked by their node degree), intermediate degree nodes (next 5–60% of ranked nodes), and low degree nodes (remaining nodes). The first group was denoted as the hub regions (nodes). Then, the proportion of the brain areas whose PE was closely associated with the states of consciousness to the total number of areas in each group was evaluated.

### 2.6. Statistical Analysis

We applied a two-side paired *t*-test for the comparison of the four states (W, L, D, and R). For the correlation analysis between PE and SMI, a one-side *t*-test was applied for each of the four states. At the individual level, the number of subjects that showed a significant correlation coefficient (*p* < 0.05) was stated. In all the hypothesis tests in our study, FDR-corrected *p*-values with less than 0.05 were considered to be significant (* *p* < 0.05; ** *p* < 0.01; *** *p* < 0.001), unless otherwise stated. 

## 3. Results

### 3.1. Propofol Differentially Suppresses Local Information in Functional Areas

**Figure 1:**
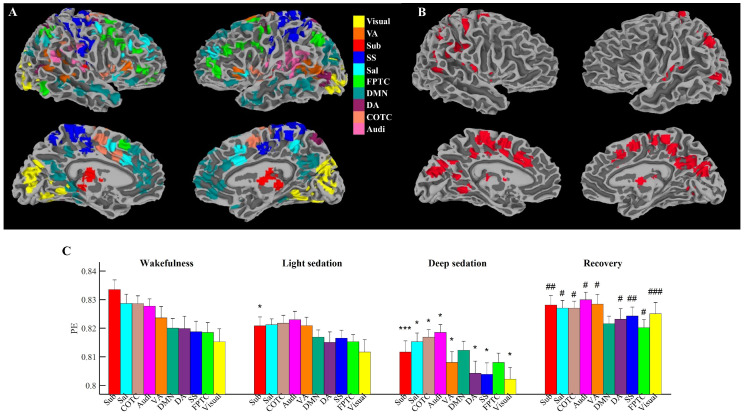
Propofol differentially affects PE of functional areas. (**A**) Spatial distribution of 10 networks; Visual: visual, VA: ventral attention, Sub: subcortical, SS: sensory/somatomotor, Sal: salience, FPTC: fronto-parietal task control, DMN: default mode network, DA: dorsal attention, COTC: cingulo-opercular task control, Audi: auditory. (**B**) Regions where PE reduced during the transition W → D and recovered during the transition D → R. (**C**) PE of 10 networks (FDR corrected *p*-values; *: different to wakefulness, #: different to deep sedation). Bar graphs are sorted by a descending order of PE of networks in wakefulness. The error bar indicates a standard error of 15 subjects.

Local information in the 226 brain areas was measured by PE, which depends on the temporal dynamics of each region (Figure 1A). Figure 1B highlights the regions where PE was reduced during the transition W → D and increased during the transition in D → R; these regions were the cuneus, posterior cingulate cortex, thalamus, medial prefrontal cortex, precuneus, superior temporal gyrus, and inferior parietal cortex. Considering the functional brain networks, the PEs of all 10 networks on average tended to decrease during the transition from the W → D state (Figure 1C). During the transition W → L, PE in Sub network decreased significantly. When comparing W to D, the PE of most networks, with the exception of the default mode network (DMN) and fronto-parietal task control (FPTC) network, significantly reduced. The PE in all networks except the DMN was enhanced during the transition D → R, along with the return of responsiveness.

### 3.2. Functional Connections Are Differentially Affected by Propofol

**Figure 2:**
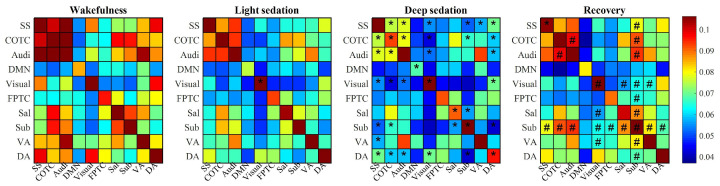
Altered SMI connectivity by propofol. The coarse-grained SMI matrix of within- and between-networks connections (10 by 10 networks) was constructed from the original 226 by 226 SMI matrix by averaging over the corresponding connections. Connections which changed significantly from wakefulness are marked as ‘*’, whereas those which changed significantly from deep sedation are marked as ‘#’. An uncorrected *p*-value < 0.05 was applied in this analysis; no significant changes were found with FDR-corrected *p*-values.

The effect of propofol on functional connectivity representing shared information among 10 networks was investigated by estimating the SMI among 226 brain regions. Firstly, a 226 by 226 SMI matrix was constructed, and within- and between-network SMI values were obtained by averaging over the corresponding connections, resulting in a 10 by 10 coarse-grained SMI matrix (Figure 2). Overall, it was found that SMI decreased by propofol induction. Some of the within- and between-networks decreased significantly from W → L and W → D transitions (uncorrected *p*-values, * *p* < 0.05 for comparison to W; # *p* < 0.05 for comparison to D). No significant changes were present when a corrected multiple comparison was performed. During the transition D → R, a substantial increase of SMI was seen in the subcortical (Sub) network. 

### 3.3. Topographic Structure of Shared Information Is Associated with States of Consciousness

Although both PE and SMI showed a tendency to reduce by propofol sedation, statistically significant global mean change was found only in PE; global PE decreased during the transition W → D and increased during the transition D → R (Figure 1A,B). 

**Figure 3:**
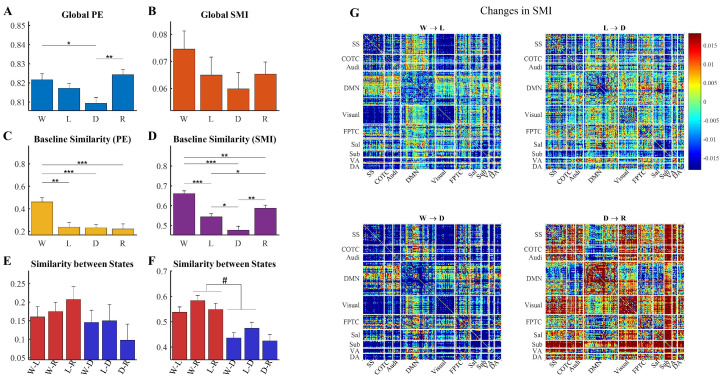
Topographic similarity of SMI distinguishes states of consciousness. (**A**,**B**) Global mean of PE (**A**) and that of SMI (**B**) across four states. (**C**) Topographic similarity of PE across four states. Spearman correlation between a reference PE vector and a PE vector for each subject for each state was calculated. The reference PE vector was obtained by average of PE vectors of 15 subjects in W state. (**D**) Topographic similarity of SMI across four states. Spearman correlation between a reference SMI matrix and a SMI matrix for each subject for each state was calculated. The reference SMI matrix was obtained by the average of the SMI matrix of 15 subjects in the W state. (**E**,**F**) Topographic similarity between states. Spearman correlation between conscious states (W vs. L, W vs. R, and L vs. R) is represented in red color, and Spearman correlation between conscious and unconscious states (W vs. D, L vs. D, and D vs. R) is represented by the color blue. ‘#’ mark indicates that the similarity between conscious states is significantly higher than the similarity between conscious and unconscious states. (**G**) Changes in SMI during the transitions, W → L (upper left), L → D (upper right), W → D (lower left), and D → R (lower right). Increased (decreased) connections during the transitions are represented in red (blue) color. The error bar indicates the standard error of 15 subjects.

The differential effect of propofol on various brain regions could be seen as a change in the topographic structure of PE and of SMI. To address this effect more directly, we assessed the topographic similarity between a reference PE vector (vectorized SMI matrix) from the W state and a PE vector (vectorized SMI matrix) for each subject for each state. Topographic similarity was assessed by using a Spearman correlation. In PE, statistical significance was found between the W vs. L, W vs. D, and W vs. R states (Figure 3C). Topographic similarity remained low in the R state and did not recover with the return of responsiveness (*p* = 0.917 for D vs. R). Topographic similarity of SMI, on the other hand, showed progressive reduction from the W through L to D state, and increased from the D to R state (Figure 3D). All six comparisons among the four states showed statistical significance, suggesting a dose-dependent effect of propofol on SMI. We also compared the topography of PE and that of SMI among the various states, without using the reference vector (Figure 3E,F). We anticipated that the topographic structures in the conscious states (W, L, and R) would be relatively similar, but that they would be distinctively different from those in the unconscious state (D). Again, the topographic similarity of different states was assessed by using the Spearman correlation. On average, the topographic similarity among conscious states (W, L, and R states) was higher than the topographic similarity between the conscious and unconscious states (W, L, or R vs. D state) for both, in PE and SMI. Statistically significant differences between the two groups (topographic similarity among the conscious states vs. topographic similarity between the conscious and unconscious states) were found only in SMI. 

### 3.4. Altered Relationship between Local and Shared Information

**Figure 4:**
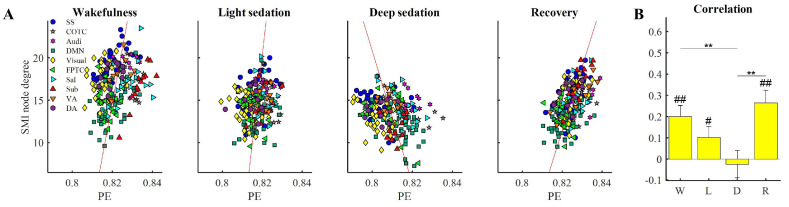
Altered relationship between PE and SMI. (**A**) Visualization of PE and SMI by using a 2-dimensional plot. To compare the 226 by 226 SMI matrix with the 226 by 1 PE vector, an SMI node degree of each region was obtained by averaging 225 corresponding SMI values. The 226 markers in each subplot correspond to PEs and SMIs of the 226 brain regions averaged across 15 subjects; different marker colors and shapes represent 10 different networks defined in Figure 1A. (**B**) The relationship between PE and SMI node degree was evaluated for each subject by assessing the Pearson correlation coefficient. Marker ‘#’ represents a significant positive relationship between the two measures in the group level (One-sided *t*-test for correlation values). Marker ‘*’ represents a significant change of correlation across four states. The error bar indicates a standard error of 15 subjects.

Because PE and SMI measure local and shared information, respectively, we were also interested in whether there was a correlation between the two measures (Figure 4). Firstly, we visualized the relationship between the two measures at the group level (Figure 4A). PE and SMI node degree values in each of 226 regions were obtained by averaging over 15 subjects. Qualitatively, the conscious states (W, L, and R) showed a positive relationship between PE and SMI, while the unconscious state (D) showed a negative relationship. Secondly, for statistical analysis, we assessed the PE-SMI relationship separately for each of the 15 subjects (Figure 4B). A significant positive correlation between PE and SMI was found in the conscious states (W, L, and R) after performing a hypothesis test with 15 correlation values collected from each of the 15 subjects; also, 11, 6, and 10 out of 15 subjects showed *p*-values for the Pearson correlation less than 0.05 in the W, L, and R states, respectively. On the other hand, D states did not reject the null hypothesis (*p* = 0.644); also, only 5 out of 15 subjects showed the significant positive correlation in D states. In addition, there was a significant reduction in the correlation values during the transition W → D, and an enhancement during the transition D → R.

### 3.5. PE Changes in High Degree Nodes Are Associated with States of Consciousness

**Figure 5:**
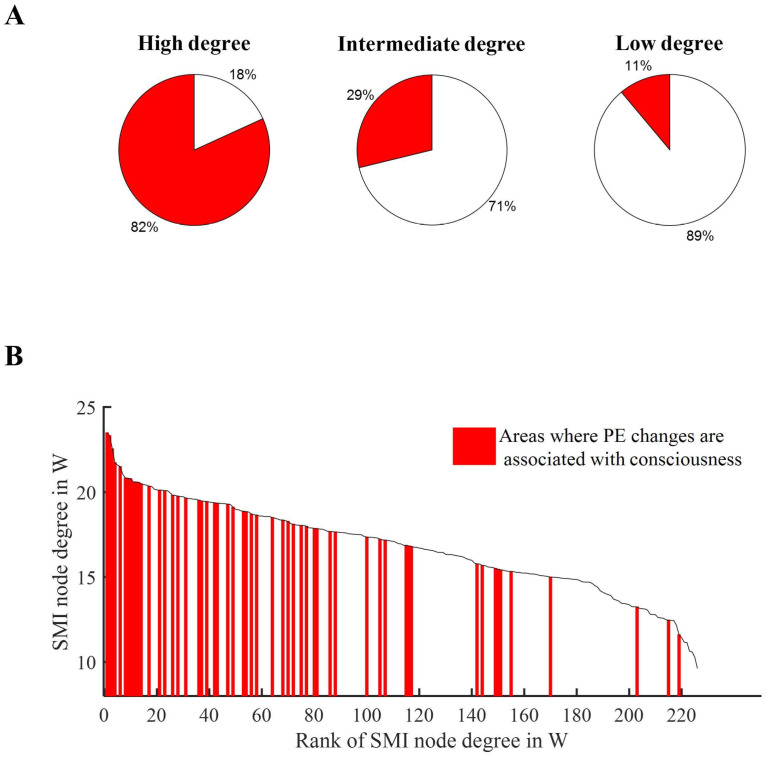
Brain areas with PE associated with loss and recovery of consciousness were those with high node degree. (**A**) Proportion of brain areas changing with state of consciousness (red areas) in three groups: high-degree nodes (top 5%), intermediate-degree nodes (5–60%), and low-degree nodes (remaining). (**B**) SMI node degree of the 226 areas ranked by node degree in the wakeful state (W). Red vertical bars represent the brain areas whose PE significantly decreased and increased during the loss and recovery of consciousness (Figure 1B). These were mostly high-degree (hub) nodes.

Lastly, we wanted to determine which of the brain areas whose PE was closely associated with the state of consciousness (Figure 1B) had a preferential distribution of SMI node degree. Figure 5A illustrates that the brain areas with PE associated with states of consciousness tended to have a high node degree in the baseline W state. The proportion of these areas were 82%, 29%, and 11% amongst the high, intermediate, and low degree groups, respectively. When plotted by the rank of the SMI node degree in the W state, the brain areas associated with states of consciousness were distributed mostly in the high-degree regime (Figure 5B). 

## 4. Discussion

We applied PE and SMI analysis to fMRI-BOLD time series and studied the changes of local and shared information during sedation with the anesthetic, propofol. Firstly, we found a decrease and an increase of PE, in the W → D and D → R transitions, respectively, consistent with human EEG studies [1,4] and an animal fMRI study [23,24]. The cuneus, posterior cingulate cortex, thalamus, medial prefrontal cortex, precuneus, superior temporal gyrus, and inferior parietal cortex showed both a decreasing pattern in W → D transition and an increasing pattern in the D → R transition, suggesting that altered dynamics in these regions may be responsible for the loss and recovery of consciousness. Secondly, functional connections were also differentially affected, resulting in a topographic reconfiguration of SMI. Thirdly, we found a positive correlation between PE and SMI in the W state. The relationship was disrupted in the D state and restored in the R state. Lastly, high-degree nodes (areas) in the W state were found to highly overlap within the brain areas whose PE changes were associated with states of consciousness.

### 4.1. Differential Effect of Propofol Sedation on Brain Regions

Altered temporal dynamics have been widely reported during anesthetic-induced unconsciousness. One way to evaluate the changes of temporal dynamics was by using measures of entropy or complexity quantifying the irregularity or unpredictability of the signal [2,4,25,26]. Despite its general success in discriminating anesthetic states, the application of entropy measures to the EEG signal did not provide information about the brain regions in which change in temporal dynamics occurs, due to the poor spatial resolution and volume conduction of EEG signals. Nevertheless, anesthetics are known to affect brain regions differentially [27,28,29,30], which is arguably important to better understand the mechanisms by which anesthetics can alter states of consciousness. This knowledge gap can be bridged by the use of fMRI with its superior spatial resolution and signal localization. In fact, a preclinical study on the fMRI signal indicated a significant effect of propofol anesthesia on spatiotemporal complexity [23,24]. However, entropy or complexity analysis on human fMRI regarding consciousness has been barely applied. To our knowledge, we applied PE for the first time in human fMRI data during propofol-induced unconsciousness. 

In this study, among the 10 pre-defined networks, the changes of PE in the Sub network were most pronounced (Figure 1C). PE in Sub network was highest in the W state, but it was substantially decreased from W to L as well as from the W to D state. PE in Sal, COTC, Audi, VA, DA, SS, and Visual networks also decreased in the W → D transition, whilst the DMN and FPTC networks did not show significant changes. PE in Sub, Sal, COTC, Audi, VA, DA, SS, and Visual networks decreased in the W → D transition and increased in the D → R transition. When considering the 226 functional areas, PE in the cuneus, posterior cingulate cortex, thalamus, medial prefrontal cortex, precuneus, superior temporal gyrus, and inferior parietal cortex showed a decreased and an increased pattern during the W → D and D → R transitions, respectively. These regions largely overlapped in the brain areas where regional cerebral blood flow significantly decreased during propofol-induced unconsciousness [31].

The differential effect of propofol appeared as a reconfiguration of topographic structure (Figure 3C,E). After propofol induction, substantial changes in topographic structure of PE were found. However, changes in topographic similarity of PE were not consistent with the dose-dependent effect of propofol; that is, there were no progressive decreases in topographic similarity from the W through L to D state and no restoration from the D to R states.

### 4.2. Topographic Structure of Functional Connectivity Characterizes States of Consciousness

A few recent studies examined the strength and topographic structure of functional connectivity in different states of consciousness [12,32,33]. One of these studies reported that in wakefulness, functional connectivity was topologically similar to anatomical structural connectivity, whereas in propofol-induced unconsciousness functional connectivity deviated from structural connectivity [32]. Based on their modeling result, together with previous studies [34,35], the authors proposed that criticality was an underlying mechanism of the similarity of functional connectivity and structural connectivity in the conscious state. They suggested that the dissimilarity of functional connectivity and structural connectivity in the unconscious state was due to a departure from critical dynamics that normally characterizes the conscious brain [36]. 

Our present findings are consistent with the conjecture of criticality as a fundamental feature of the conscious brain, and they also provide new insights. Specifically, we compared the topographic structure of SMI in various states to the topographic structure of the SMI averaged across all subjects during the W state as a reference. The topographical similarity with this reference showed a progressive decrease in the L and D state, and an increase in the R state (Figure 3D). Importantly, the difference between the L and D states indicates a significant change of topographic structure during the transition from consciousness to unconsciousness. Furthermore, the topographic structure in the unconscious state (D), was distinct from all conscious states (W, L, and R), while the latter three had similar topographic structures with each other (Figure 3F). 

### 4.3. Propofol Sedation Disrupts the Relationship between Local and Shared Information

In addition to studying changes in PE and SMI during anesthesia, the relationship between PE and SMI and its changes across different states were also explored. Firstly, we found a positive relationship between PE and SMI in the W state (Figure 4). This suggests that the brain regions with substantial local information (high PE) may be more likely to interact with each other, resulting in relatively high shared information (high SMI). The qualitatively similar results obtained with normalized SMI confirmed that the higher values of shared information could not be explained by higher local information of the interacting regions. Interestingly, the relationship between PE and SMI was disrupted in the unconscious state D. We conjecture that the positive correlation between local and shared information may underlie natural information-processing in the conscious brain, and that the disruption of this relationship may indicate impaired cognitive function. In other words, the existence of locally informative but segregated regions can be understood as an indication of an abnormal state, as seen during propofol-induced unconsciousness in our study (Figure 4A).

In Figure 5, we demonstrated that loss (and recovery) of consciousness is related to a suppression (enhancement) of local information in hub regions. Considering the pivotal role of hubs in information integration and disintegration, targeting hub regions would be an efficient way for anesthetics to disrupt most of our cognitive functions, as well as consciousness. However, it is unclear whether the result comes from a direct preferential influence of the anesthetic to the hub regions or whether it represents a secondary outcome from an unknown network-level mechanism [37]. In addition, it should be tested whether the disrupted information-processing in the hub region is also associated with anesthetic-induced unconsciousness with distinct molecular actions and pathologically-induced unconsciousness. For the latter, an initial failure (suppression of information) may not be in the hub regions, but it can eventually lead to a hub failure, leading to loss of consciousness. Therefore, understanding the role of the hub structure in information-processing and its modification is crucial to better understanding the network-level mechanisms of anesthetic-induced unconsciousness.

Recently, Huang et al. [12] suggested that enhanced synchrony in local circuits during propofol sedation breaks down distant functional connectivity. In our study, hyper-synchronization in a local area might appear as suppressed local information, because synchronous signals give rise to a rhythmic, regular pattern of activity with low information content. Another recent study demonstrated that the reduction of transfer entropy in isoflurane anesthesia could be attributed to a suppression of source entropy in a local area [38]. This conclusion fits the fact that the synaptic targets of anesthetics are likely located in local circuits [39,40]. It is also noteworthy that current devices for monitoring anesthetic depth utilize single-channel EEG signals without considering the functional connections between distant regions. In our study, the changes of local information appear to be more pronounced than those of shared information. Thus, a change of global mean value was seen in PE, but not in SMI; the PE of hub nodes was significantly reduced, whereas the SMI of hub nodes was essentially unchanged. When multiple comparisons were used, SMI values were insignificantly altered whereas the PEs of many regions were significantly affected. However, although SMI changes across states were not consistent, the SMI topography was statistically distinctive across the four states; the topographic similarity of SMI was the only measure that could statistically differentiate all of the four states in our data (Figure 3D). There would be a need for systematic investigation of the temporal precedence or the causal relationship between local and shared information, as well as to know which of these are responsible for unconsciousness.

### 4.4. Limitations of the Study

Our study has several limitations. Firstly, we defined unconsciousness as a loss of behavioral responsiveness to verbal command (OAAS score 2-1). Therefore, participants may have had some form of conscious experience even during the D state, while not responding to external stimuli [41,42,43]. In the future, a more sophisticated experimental design may be necessary to distinguish between conscious experience and responsiveness. Secondly, it should be kept in mind that we applied the information theoretic measures to fMRI data, not to individual neurons. Therefore, the notion of local and shared information in our study should be carefully interpreted. Thirdly, we recognize the possibility that propofol may have altered cerebral hemodynamics through a direct effect on the cerebral vasculature or cerebral metabolic activity in addition to its effect on neural activity. It is well-known that, at surgical level, propofol decreases the cortical metabolic rate [44]. However, during light to moderate sedation, the effects of propofol on cerebral hemodynamics and metabolic activity are minimal [31,45], while changes in neural activity are substantial [46,47]. Therefore, it is likely that our findings reflect a primary effect of propofol on neural activity rather than those secondary to vascular or metabolic activity. Fourthly, we did not examine deep, surgical levels of anesthesia. A recent study reported that the power spectrum of fMRI BOLD signal exhibits a flat distribution similar to that of white Gaussian noise at high propofol concentration (4.0 μg/mL) corresponding to surgical anesthesia [48]. This finding suggests that in deep anesthesia PE might be high compared to that in wakefulness, as observed in EEG [3,4]. During the transition from sedation to deep anesthesia, many quantities exhibit a biphasic effect; that is, an initial decrease (or increase) of the entropy variable is followed by an increase (or decrease) at higher anesthetic concentration levels [5,48,49]. However, the enhancement of entropy measures at high anesthetic concentration levels may result from an increased randomness of neural dynamics (i.e., prolonged suppression periods), not from an augmentation of local information [4]. It is possible that the monotonic decrease of SMI topographic similarity would follow the level of sedation in a dose-dependent manner, even in deep anesthesia (Figure 3D). Future studies should validate this hypothesis.

## 5. Conclusions

By applying ordinal pattern analysis to fMRI signals during different states of consciousness, we found that that the anesthetic propofol at light to deep sedative doses (1) has a differential effect on brain regions; (2) induces a topographical reorganization of shared information; (3) breaks down the positive relationship between local and shared information; and (4) disrupts local information preferentially in the hub regions of brain networks. These findings advance our understanding of the mechanism of anesthetic-induced unconsciousness.
